# Xenon triggers pro-inflammatory effects and suppresses the anti-inflammatory response compared to sevoflurane in patients undergoing cardiac surgery

**DOI:** 10.1186/s13054-015-1082-7

**Published:** 2015-10-15

**Authors:** Thomas Breuer, Christoph Emontzpohl, Mark Coburn, Carina Benstoem, Rolf Rossaint, Gernot Marx, Gereon Schälte, Juergen Bernhagen, Christian S. Bruells, Andreas Goetzenich, Christian Stoppe

**Affiliations:** Department of Anaesthesiology, University Hospital of the RWTH Aachen, Pauwelsstr. 30, 52074 Aachen, Germany; Department of Thoracic and Cardiovascular Surgery, University Hospital of the RWTH Aachen, Aachen, Germany; Department of Intensive and Intermediate Care, University Hospital of the RWTH Aachen, Pauwelsstr. 30, 52074 Aachen, Germany; Institute of Biochemistry and Molecular Cell Biology, University Hospital, RWTH Aachen University, Pauwelsstr. 30, 52074 Aachen, Germany

## Abstract

**Introduction:**

Cardiac surgery encompasses various stimuli that trigger pro-inflammatory mediators, reactive oxygen species and mobilization of leucocytes. The aim of this study was to evaluate the effect of xenon on the inflammatory response during cardiac surgery.

**Methods:**

This randomized trial enrolled 30 patients who underwent elective on-pump coronary-artery bypass grafting in balanced anaesthesia of either xenon or sevoflurane. For this secondary analysis, blood samples were drawn prior to the operation, intra-operatively and on the first post-operative day to measure the pro- and anti-inflammatory cytokines interleukin-6 (IL-6), interleukin-8/C-X-C motif ligand 8 (IL-8/CXCL8), and interleukin-10 (IL-10). Chemokines such as C-X-C motif ligand 12/ stromal cell-derived factor-1α (CXCL12/SDF-1α) and macrophage migration inhibitory factor (MIF) were measured to characterize xenon’s perioperative inflammatory profile and its impact on migration of peripheral blood mononuclear cells (PBMC).

**Results:**

Xenon enhanced the postoperative increase of IL-6 compared to sevoflurane (Xenon: 90.7 versus sevoflurane: 33.7 pg/ml; p = 0.035) and attenuated the increase of IL-10 (Xenon: 127.9 versus sevoflurane: 548.3 pg/ml; p = 0.028). Both groups demonstrated a comparable intraoperative increase of oxidative stress (intra-OP: p = 0.29; post-OP: p = 0.65). While both groups showed an intraoperative increase of the cardioprotective mediators MIF and CXCL12/SDF-1α, only MIF levels decreased in the xenon group on the first postoperative day (50.0 ng/ml compared to 23.3 ng/ml; p = 0.012), whereas it remained elevated after sevoflurane anaesthesia (58.3 ng/ml to 53.6 ng/ml). Effects of patients’ serum on chemotactic migration of peripheral mononuclear blood cells taken from healthy volunteers indicated a tendency towards enhanced migration after sevoflurane anaesthesia (p = 0.07).

**Conclusions:**

Compared to sevoflurane, balanced xenon anaesthesia triggers pro-inflammatory effects and suppresses the anti-inflammatory response in cardiac surgery patients even though the clinical significance remains unknown.

**Trial registration:**

This clinical trial was approved by the European Medicines Agency (EudraCT-number: 2010-023942-63) and at ClinicalTrials.gov (NCT01285271; first received: January 24, 2011).

**Electronic supplementary material:**

The online version of this article (doi:10.1186/s13054-015-1082-7) contains supplementary material, which is available to authorized users.

## Introduction

Xenon’s well-known neuro- and cardioprotective properties render this noble gas an attractive alternative to conventional volatile anaesthetics, which has been intensively investigated in a variety of experimental and clinical trials [1–5]. Previous studies controversially discussed xenon’s effects on the inflammatory response, whereas the underlying mechanisms remained elusive [6, 7]. In this context, de Rossi and co-workers previously demonstrated anti-inflammatory properties of xenon, which were supposed to result from increased removal of selectin PSGL-1 and L-selectin [7]. In addition, xenon was suggested to limit myocardial and brain injury through inhibition of the N-methyl-d-aspartate (NMDA) receptor. This is of particular relevance during excessive activation after ischaemia and reperfusion and frequently results in glutamate excitotoxicity [8, 9]. In contrast, only a few experimental studies have demonstrated xenon’s pro-inflammatory properties, which have been attributed to its leucocyte- and platelet-activating properties [6] and/or influence on Ca^2+^ homeostasis [5].

The use of cardiopulmonary bypass (CPB) is recognized as a potent stimulus for the release of inflammatory mediators, reactive oxygen species and mobilization of leucocytes that contribute to cardiac dysfunction [10, 11] and organ injury [12–14], which is of particular relevance for patients undergoing cardiac surgery [15]. In this context, the influence of anaesthetics on the inflammatory response and oxidative stress has increasingly been considered [16, 17]. Emerging evidence indicates that xenon has beneficial effects on global hemodynamic performance, myocardial contractility and coronary blood flow [18–21]. In addition previous studies demonstrated that xenon augments myocardial recovery and limits the extent of myocardial injury in different animal models, which may be of particular relevance for patients undergoing cardiac surgery [22, 23].

To further characterize xenon’s influence on the inflammatory response, we performed a secondary analysis of a recently published randomized controlled trial that evaluated the safety and feasibility of balanced xenon anaesthesia in cardiac surgery patients [24]. As the clinical data did not support xenon’s superiority compared to the well-established volatile sevoflurane, we hypothesized that underlying mechanisms may be due to xenon-induced pro-inflammatory effects.

## Materials and methods

### Study design and patients

The present study is a predefined secondary analysis of a randomized, single-blinded, controlled clinical trial approved by the local institutional review board (Ethics Committee of the RWTH Aachen University, Faculty of Medicine, Aachen, Germany; ethic vote: 10–017), the German Federal Drug Administration (BfArM), and registered at the European Medicines Agency (EudraCT-number: 2010-023942-63) and at ClinicalTrials.gov (NCT01285271). In total, 30 patients scheduled for elective cardiac surgery with the use of cardiopulmonary bypass were included in this prospective single-centre study. Exclusion criteria were emergency operations, known or suspected pregnancy, patients’ age less than 18 years, and failure to obtain informed consent. Peripheral mononuclear blood cells were taken from healthy volunteers for performance of migration assays (ethic vote: EK 191/14).

For cardiopulmonary bypass a circuit (Stockert s5, Sorin Group Germany, Munich, Germany) with moderate hypothermia (32–34 °C) was used. Antegrade infusion of cold crystalloid cardioplegic solution (CustodiolTM, Koehler Chemie, Alsbach-Haehnlein, Germany) induced cardiac arrest. A non-pulsatile pump flow of 2.2 litre min^−1^ m^−2^ was used for extracorporeal circulation.

Details of the study were published elsewhere [25]. In brief, patients were randomized to receive either balanced anaesthesia using xenon (end-expiratory concentrations of 45–50 % vol) or sevoflurane (end-expiratory concentrations of 1.0–1.4 % volume) each combined with continuous infusion of sufentanil (0.5–1.5 μg kg^−1^ h^−1^). To enable a comparable depth of anaesthesia, continuous monitoring of the bispectral index (BIS, Convidien, Dublin, Ireland) was performed in all patients. After surgery, all patients were transferred to the intensive care unit (ICU) and the postoperative treatment was standardized according to our institutional guidelines.

In order to analyse the complex immune response to xenon and sevoflurane exposure in cardiac surgery patients we first measured the clinically common cytokine and acute phase protein interleukin-6 (IL-6) as well as IL-8/CXCL8 and their anti-inflammatory counterpart IL-10, followed by the analysis of oxidation-reduction potential (ORP) to account for inflammation-aggravating effects of oxidative stress. To further investigate the chemotactic properties of both anaesthetic gases we measured stromal cell-derived factor-1α (SDF-1α) and macrophage migration inhibitory factor (MIF), and performed peripheral blood mononuclear cell (PBMC) migration assays.

### Laboratory assessments

In addition to clinical routine measurements, serum samples from patients were drawn directly before induction of anaesthesia (pre-OP), immediately before termination of surgery (intra-OP) and 24 h postoperatively (post-OP). We measured cytokine concentrations, determined serum oxidation-reduction potential (ORP) as an indicator of oxidative stress, and investigated serum influence on the migration of PBMCs. The blood samples were immediately centrifuged (3,000 rpm, 10 minutes) and the supernatant transferred to cryotubes. The serum samples were subsequently stored at −80 °C until analysis in 2013 and 2014.

### Serum cytokine concentration

Serum concentrations of SDF-1α/CXCL12, IL-6, IL-8/CXCL8 and IL-10 were determined with commercially available ELISA assays (CXCL12, IL-6, IL-8, IL-10; R&D Systems, Wiesbaden-Nordenstadt, Germany) according to the manufacturer’s instructions. Serum levels of MIF were assessed using an ELISA technique as previously described [26], using capture antibody MAB289 and detection antibody BAF289 (both R&D Systems, Wiesbaden-Nordenstadt, Germany).

### Measurement of the oxidation-reduction potential in serum samples

The measurement of ORP provides a reliable method to assess the balance between total pro-oxidants and anti-oxidants in the blood [27]. A higher static ORP is indicative of oxidative stress. Taking into account that cardiac surgery is a contributing source of oxidative stress [28] we measured the static ORP of the serum samples as previously described [29, 30].

### Isolation of PBMCs from healthy volunteers for in vitro culture

All cell culture activity assays were performed under sterile conditions in a laminar flow hood. The cells were cultured in a CO_2_-incubator at 37 °C and 5 % CO_2_. Mononuclear cells were obtained from five buffy coats, which were received from healthy volunteers (n = 5) after informed consent in accordance with the local ethics committee (Ethics Committee of the RWTH Aachen University, Faculty of Medicine, Aachen, Germany; ethic vote: EK 191/14). The cells were separated by Ficoll density gradient centrifugation (GE Healthcare, Chalfont, UK) and subsequently plated on cell culture dishes (Greiner Bio-One, Kremsmuenster, Austria) and cultured in medium (RPMI 1640), enriched with 10 % fetal calf serum, 1 % Penicillin/Streptomycin and 1 % non-essential amino acids (all from Life Technologies Carlsbad, USA). The medium was changed after 3 days to remove non-adherent cells and cells were harvested on day 4.

For detachment of adherent cells prior to migration experiments, the medium was removed and the cells were washed with phosphate-buffered saline (PBS). Afterwards, a cell scraper (Corning Inc., Tewksbury, USA) was used to detach the cells, which were subsequently re-suspended in RPMI 1640 serum-starved medium containing only 0.5 % bovine serum albumin (BSA, Roth, Karlsruhe, Germany). The cells were washed with PBS and finally, the solution was centrifuged for 15 minutes at 1,250 rpm and the pellet was re-suspended in RPMI 1640 serum-starved medium containing 0.5 % BSA (serum-starved medium, Sigma Aldrich, St. Louis, MO, USA).

### PBMC migration assays

In order to reveal the influence of different chemokines within the serum samples on inflammatory cells, PBMC migration assays were performed using a Transwell chamber (Corning Inc., Tewksbury, USA) in a 96-well plate format and cell culture inserts containing filters with a pore-size of 5 μm (Corning Inc., Tewksbury, USA). The cells were detached as described above and counted. For the migration assay, 50.000 cells per well (in 75 μl medium) were used. Lower chambers contained serum samples (1:5 dilution) in RPMI 1640 medium containing 0.5 % BSA. PBMCs in the same medium were placed into the upper chamber of each well.

A second migration assay was performed to investigate the effect of immunosuppressive factors within the serum samples. PBMCs were first divided into Eppendorf tubes, each containing one serum sample (1:2 dilution with RPMI 1640 serum-starved medium) and incubated for 6 h. Subsequently, the serum samples were further diluted (1:5) and 50.000 cells of each Eppendorf tube were seeded into one migration chamber. Afterwards, all cells were allowed to migrate towards 100 ng/ml recombinant MCP-1 (Peprotech, Rocky Hill, USA).

In both cases, after three hours of migration at 37 °C and 5 % CO_2_, all cell culture inserts were removed. To optimize the counting conditions, migrated cells were fixed and stained with Hoechst dye (ImmunoChemistry Technologies, LLC, Bloomington, USA) diluted in 3.6 % paraformaldehyde (PFA) (1:1000). Finally, the fixed cells were incubated overnight.

Pictures were taken under a microscope (×100 magnification) the next morning and migrated cells were counted by an independent team member blinded to the study using the semi-automated software ImageJ (National Institutes of Health, Maryland, USA). Apart from diminishing the background and optimizing counting conditions, the software allows marking of the cells and adds them automatically onto a tally sheet. To guarantee that counting conditions were the same in each individual experiment, pictures were taken of the same position within the migration chambers in each individual experiment.

### Statistical analysis

All data were statistically analysed using a commercially available software package (GraphPad Prism 6.0, Graphpad Software Inc., San Diego, CA, USA; SPSS 21, IBM, USA). All data were tested for normal distribution with the Shapiro–Wilk’s test. Given the explorative nature of this pre-planned post-hoc analysis, normally distributed data from single measurements were compared between the groups at single time points using Student’s *t* test (two groups), which was adjusted for multiple measurements, in accordance with our statistician’s advice. Non-parametric single measurements were compared using the Mann–Whitney *U* test. Proportions were compared using the Chi-square test. In all cases, a level of *p* <0.05 was considered statistically significant.

## Results

### Baseline characteristics

Baseline characteristics of enrolled patients are reported elsewhere [24]. No significant differences between the groups were detected prior to surgery and patients included reflect a representative cohort of cardiac surgery patients. The time course of BIS values was comparable between both groups during the entire observation period.

### Xenon stimulates the perioperative increase in IL-6, but not IL-8, IL-10 and oxidative stress

**Fig. 1:**
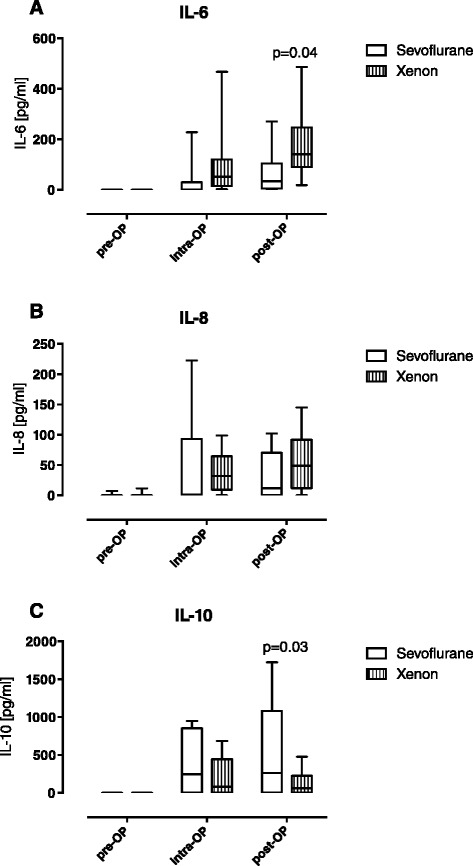
Perioperative time course of interleukin (*IL*)-6 (IL-6) (**a**), IL-8 (**b**) and IL-10 (**c**). Circulating serum levels of IL-6, IL-8, and IL-10 were measured perioperatively in serum samples of patients who underwent cardiac surgery. Values are depicted in pg/ml. IL-6, IL-8 and IL-10 increased during the surgical intervention. While IL-6 levels increased postoperatively in the xenon group compared to the sevoflurane group, IL-10 levels only increased significantly in the sevoflurane group during surgery. Serum levels of IL-8 levels did not differ between both groups during the observation. Data are shown as *boxplots* with means and maximal to minimal values (*p* values are indicated within the figure). *Pre-OP* baseline, before induction of anaesthesia, *intra-op* immediately before termination of surgery, *post-OP* 24 h after surgery

To characterize the influence of xenon versus sevoflurane on the inflammatory response, we measured serum levels of well-known inflammatory cytokines and oxidative stress perioperatively. Circulating levels of IL-6 decreased significantly in the xenon group compared to the sevoflurane group after the end of surgery (xenon (Xe) 90.7 pg/ml vs. sevoflurane (Sev) 33.7 pg/ml; *p* = 0.035, Fig. 1a). In contrast, we did not find any significant differences in IL-8 release between both groups during the entire observation period (Fig. 1b). In terms of the anti-inflammatory response, serum levels of IL-10 increased continuously in the sevoflurane group, whereas there were no major changes in levels of IL-10 after xenon exposure (127.9 vs. 548.3 pg/ml; *p* = 0.028, Fig. 1c). As oxidative stress, is known to amplify the perioperative immune response [31], redox balance was assessed in the included patients. No significant differences in oxidative stress were detected between the groups during the study (Additional file 1).

### Xenon reduces the postoperative serum levels of MIF but does not affect perioperative serum levels of SDF-1α

**Fig. 2:**
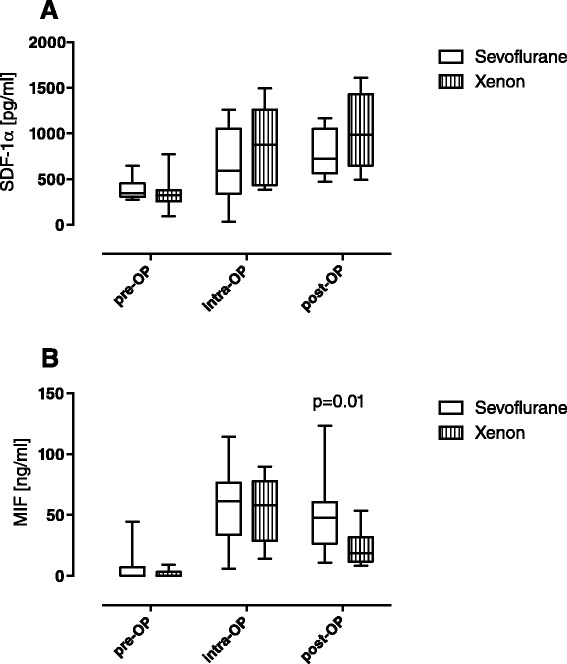
**a** Perioperative time course of CXCL12/stromal cell-derived factor 1α (*SDF-1α*) serum concentrations. Measurement of CXCL12/SDF-1α levels in the serum of patients who underwent cardiac surgery. Values are depicted in pg/ml. CXCL12 levels increased in both groups during surgical intervention. However, there were no significant differences between groups. Shown as *boxplots* with means and maximal to minimal values (*p* values are indicated). *Pre-OP* baseline, before induction of anaesthesia, *intra-op* immediately before termination of surgery, *post-OP* 24 h after surgery. **b** Perioperative time course of macrophage migration inhibitory factor (*MIF*) serum concentrations. The measurement of perioperative circulating MIF levels in cardiac surgery patients was performed as described previously [25]. Values are depicted in ng/ml. There was a strong intraoperative increase in serum MIF levels in both groups, which decreased again postoperatively. Postoperative measured MIF levels were significantly reduced after xenon anaesthesia compared to sevoflurane. Data are shown as *interleaved boxes* with means and maximal to minimal values (*p* values are indicated)

Recent data indicate a cardioprotective role during acute myocardial ischaemia/reperfusion [32, 33] and in patients undergoing cardiac surgery [25, 26]. Therefore we compared the perioperative profile of MIF and demonstrated a significant intraoperative increase in MIF in both groups, whereas serum levels in the xenon group were significantly lower on the first postoperative day in comparison to the sevoflurane group (Xe 23.3 pg/ml vs. Sev 53.6 pg/ml; *p* = 0.012; Fig. 2b).

SDF-1α is crucially involved in trafficking of immune cells during inflammation. We therefore analysed circulating levels of SDF-1α. Serum levels did not differ significantly between the groups during the intervention (*p* = 0.157, Fig. 2a).

### Xenon does not facilitate the chemotactic migration of peripheral blood mononuclear cells (PBMCs)

**Fig. 3:**
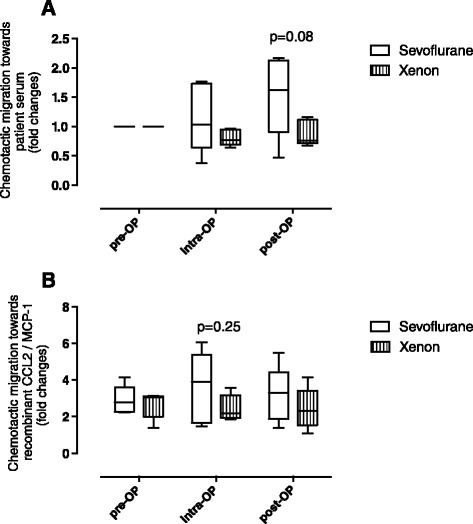
Migration assay of peripheral blood mononuclear cells (PBMCs) in serum samples of cardiac surgery patients after xenon or sevoflurane anaesthesia. **a** In vitro migration of PBMCs is increased by serum samples of patients after sevoflurane anaesthesia. Extent of PBMC migration (received from healthy volunteers) towards serum samples from cardiac surgery patients is demonstrated in both groups. While PBMC migration towards serum samples was increased during surgery in the sevoflurane group, PBMC migration was not affected in serum samples from the xenon group. Data are shown in *boxplots* with means and maximal to minimal values (*p* values are indicated). *Pre-OP* baseline, before induction of anaesthesia, *intra-op* immediately before termination of surgery, *post-OP* 24 h after surgery. **b** Serum samples from patients after xenon anaesthesia show no chemokinetic effect on PBMCs. Migration of PBMCs from healthy volunteers, which were pre-incubated in serum samples elicited by recombinant CCL2/monocyte chemoattractant protein-1 (MCP-1), is demonstrated. No significant difference was measured between the groups during surgery. Data are shown as *boxplots* with means and maximal to minimal values (*p* values are indicated)

In extension, we investigated potential anaesthesia-induced effects on the recruitment of immune cells, which are known to further enhance pro-inflammatory responses after myocardial ischaemia. Serum samples taken intraoperatively or postoperatively from the xenon group did not influence PBMC migration when compared to the effect of preoperatively drawn serum samples as control. In contrast, serum samples from patients treated with sevoflurane triggered a measurable increase in the chemotactic response of PBMCs (Fig. 3a).

Aesthetics may not only directly lead to the release of pro-chemotactic molecules but may also indirectly affect the migratory behaviour of circulating inflammatory cells, including contributions to chemokinetic effects. To study such a potential influence on circulating inflammatory cells in the blood of cardiac surgery patients, PBMCs from healthy volunteers were pre-incubated with randomly selected anaesthetic-conditioned serum samples from both groups to mimic effects on chemokinesis, e.g., activation or downregulation or migration-competent receptors, or desensitization of signalling pathways. Afterwards, a chemotaxis assay was performed with the PBMCs subjected to a chemotactic gradient of recombinant MCP-1/CCL-2 placed in the lower chamber of the migration device. Sevoflurane-preconditioned PBMCs trended towards elevated migration activity, whereas PBMC pre-treatment with xenon-conditioning postoperatively had no effect at all on migration behaviour (Fig. 3b). Together, while not statistically significant at *p* <0.05, these findings indicated that sevoflurane promotes the migration of circulating mononuclear cells by both chemotactic and chemokinetic mechanisms.

### Significance of xenon anaesthesia in relation to outcome of patients

For the clinical significance of xenon in terms of outcome, there were no significant differences between the two interventional groups, as has already been reported in more detail elsewhere [24].

## Discussion

The present study is the first that highlights the influence of xenon anaesthesia on the inflammatory response in patients undergoing cardiac surgery. Our data demonstrate that balanced xenon anaesthesia triggers pro-inflammatory effects and suppresses the anti-inflammatory response compared to the well-known anaesthetic, sevoflurane, in the complex clinical setting of cardiac surgery.

Recently, the first randomized controlled trial demonstrated the feasibility and safety of balanced xenon anaesthesia for cardiac surgery [24]. In terms of the significance of xenon in relation to clinical outcome, previous data did not confirm the hypothesis that xenon may represent superior characteristics compared to the well-known anaesthetic, sevoflurane, and the underlying mechanisms remained unknown. As the inflammatory response is widely known to determine the extent of organ dysfunction after surgery, we compared the inflammatory profile between patients after balanced xenon or sevoflurane anaesthesia. Interestingly, we found significantly higher IL-6 levels after xenon anaesthesia and the intraoperative release of anti-inflammatory IL-10 was significantly reduced in cardiac surgery patients when compared to sevoflurane. While knowledge about xenon-induced effects on the inflammatory response during cardiac surgery is limited to experimental data, Kawamura and co-workers demonstrated that sevoflurane is able to suppress the production of IL-6, but not IL-10 [34]. Furthermore, it was shown that the addition of sevoflurane to blood cardioplegia resulted in a reduced activity of neutrophils [35], thereby showing an anti-inflammatory effect [36].

In addition to these well-known cytokines, we measured serum levels of the pleiotropic cytokine MIF, which has chemokine-like functions and recently was demonstrated to provide cardioprotective effects during acute myocardial ischaemia/reperfusion [32, 33]. MIF serum concentrations showed a comparable intraoperative increase in both groups, whereas MIF levels remained significantly elevated in patients after sevoflurane anaesthesia until the 1^st^ postoperative day. As previous studies reported on the anti-inflammatory and thus, organoprotective effects of MIF during cardiac surgery [25, 26], it remains speculative whether the significantly decreased MIF levels in the xenon group may reduce the defence mechanisms in cardiac surgery patients compared to sevoflurane.

In view of the diverging inflammatory response, we next investigated the overall effect of patients’ serum samples, including the highly complex cytokine cocktail, on migration of immune cells. We found a tendency of a higher migration of PBMCs towards serum samples obtained from sevoflurane-treated patients compared to xenon. In vivo, migration of PBMCs is crucial for clearance of dead cells, tissues and subsequent regeneration. In this context, significant suppression of post-ischaemic inflammation is associated with detrimental consequences [37]. Therefore, the reduced migration of PBMCs in the xenon group may significantly affect the essential repair mechanisms after cardiac surgery.

To identify the potential key players for recruitment of PBMCs in both groups we measured serum levels of IL-8 and SDF-1, which are well-known chemokines in the inflammatory response for the trafficking of immune cells to the damaged tissue [38, 39]. Of note, recent studies indicate that SDF-1α provides pro-inflammatory properties, which may turn to anti-inflammatory function after leukocyte invasion [40]. However, we did not find any significant differences in the perioperative profile of SDF-1α or IL-8 levels between xenon and sevoflurane exposure, indicating that (non-classical) factors other than SDF-1α or IL-8 may be responsible for the post-ischaemic recruitment of PBMCs. In this context recent data demonstrate that the effect of SDF is abolished by heparin, both on the chemokine and receptor side of the signalling pathway [41]. As cardiac surgery patients receive high-dose heparin intraoperatively this effect may significantly influence the effect on migration of PBMCs in the present study.

Last, we compared the extent of oxidative stress by measurement of the redox balance in both treatment groups. Oxidative stress may lead to myocardial injury after reperfusion [42]. However, the extent of oxidative stress measured by ORP did not reveal any significant differences between xenon and sevoflurane exposure, indicating that neither sevoflurane nor xenon is able to significantly reduce the well-known intraoperative increase in oxidative stress [43, 44].

The present findings are in apparent contrast to previous results, which showed comparable effects on the immune system after xenon or sevoflurane anaesthesia in patients during general abdominal surgery [45]. However, these contrary findings should be considered cautiously in the light of different clinical settings, which are characterized by a significantly different inflammatory profile. While general surgery is known to stimulate only a mild systemic immune response, cardiac surgery frequently triggers an overwhelming perioperative immune reaction that frequently leads to the development of postoperative organ dysfunction [46].

### Limitations

We acknowledge that the present sub-study has several limitations, which may mitigate the significance of the present findings. First, this study was performed in a small patient cohort and therefore may not be adequately powered for the measurements performed. Therefore, we concede that the present data should be interpreted cautiously. However, the presented findings were received from a randomized, single-blinded, controlled clinical trial and thus, may be considered hypothesis-generating.

Second, we realize that the present analysis only focused on cytokines and chemokines, which were considered as most likely to be crucially involved in inflammatory response, with significance in relation to the clinical outcome of patients. Due to the limited quantity of serum samples we had to focus on a panel of crucial mediators. Additional analyses are encouraged to provide further insight into in vivo mechanisms induced by xenon, to enable a more comprehensive understanding of the effects of xenon and the frequently observed discrepancy between experimental and clinical studies [6, 7, 47].

Third, as sevoflurane is known to have cardioprotective properties, we assume that comparison to an anaesthetic with neutral effects would have been desirable. However neither propofol nor an alternative volatile anaesthetic would comply with these requirements. Last, the absolute serum levels should be interpreted cautiously, as storage might have influenced the detection of cytokine levels. However, duration of storage did not differ between the treatment groups.

Furthermore, it remains elusive whether xenon aggravates the pro-inflammatory response or whether the mediated effects of xenon are inferior to the well-known properties of sevoflurane in the setting of cardiac surgery. We presume that the pro-inflammatory properties of xenon may have counterbalanced its well-known promising effects, which have been repeatedly demonstrated in the past [1–5].

## Conclusion

The present study demonstrates that balanced xenon anaesthesia aggravates the inflammatory response and increases the intraoperative release of the pro-inflammatory cytokine IL-6, whereas the secretion of anti-inflammatory cytokines IL-10 and MIF was suppressed during cardiac surgery in comparison to sevoflurane. Additional large-scale prospective studies are needed to evaluate the role of xenon as a potential alternative to sevoflurane in the setting of cardiac surgery.

## Key messages

- Balanced xenon anaesthesia aggravates the inflammatory response in cardiac surgery patients
- Xenon stimulates the intraoperative release of the pro-inflammatory cytokine IL-6 but not IL-8
- Xenon suppresses the increase of anti-inflammatory cytokines IL-10 and MIF during cardiac surgery
- Compared to the well-established anaesthetic sevoflurane, xenon does not alter intraoperatively measured oxidative stress
- Xenon does not facilitate the well-known chemotactic migration of PBMCs during cardiac surgery

## Additional file

Additional file 1:
**Measurement of intra- and postoperative redox balance.** The intra- and postoperative redox balance was measured by static oxidation-reduction potential (*ORP*) in the serum samples and given in millivolts (mV). On comparison the xenon and sevoflurane groups had a comparable time course for ORP, indicating that both anaesthetics have the same influence on oxidative stress in cardiac surgery patients. Data are mean values ± SD. *Pre-OP* baseline, before induction of anaesthesia, *intra-op* immediately before termination of surgery, *post-OP* 24 h after surgery. (PDF 39 kb)

## Abbreviations

BfArM: Bundesinstitut für Arzneimittel und Medizinprodukte, Germany
BIS: bispectral index
BSA: bovine serum albumin
CPB: cardiopulmonary bypass
CXCL12/SDF-1α: stromal cell-derived factor 1α
ELISA: enzyme-linked immunosorbent assay
ICU: Intensive Care Unit
IL: interleukin
intra-OP: time point immediately before termination of surgery
MIF: macrophage migration inhibitory factor
MV: millivolts
NMDA: N-methyl-d-aspartate
ORP: oxidation-reduction potential
PBMC: peripheral blood mononuclear cells
PBS: phosphate-buffered saline
post-OP: 24 hours after surgery
pre-OP: baseline; time point before induction of anaesthesia
rpm: revolutions per minute
Sev: sevoflurane
Xe: xenon
