# Supplementary figures and images for: Xenon triggers pro-inflammatory effects and suppresses the anti-inflammatory response compared to sevoflurane in patients undergoing cardiac surgery
Source: Crit Care. 2015 Oct 15;19:365. doi: 10.1186/s13054-015-1082-7 (PMC4607103; doi:10.1186/s13054-015-1082-7)

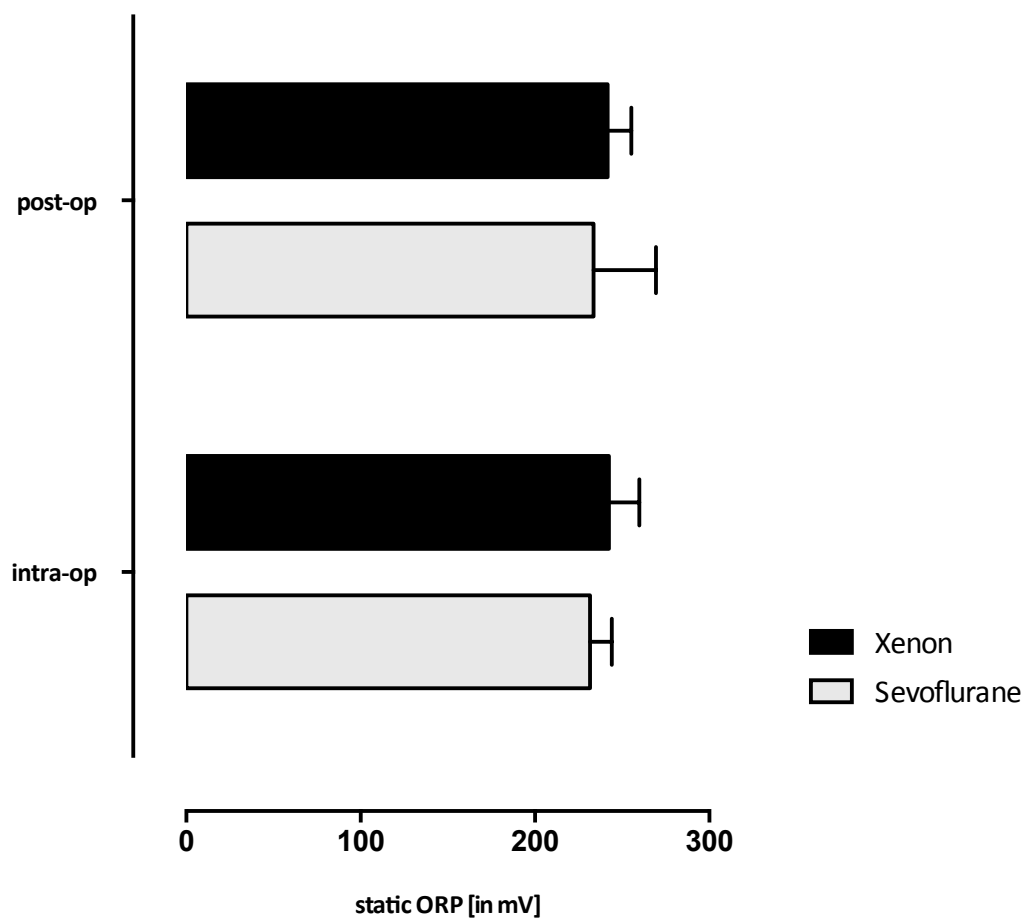

Supplement: Additional file 1: — Measurement of intra- and postoperative redox balance. The intra- and postoperative redox balance was measured by static oxidation-reduction potential (ORP) in the serum samples and given in millivolts (mV). On comparison the xenon and sevoflurane groups had a comparable time course for ORP, indicating that both anaesthetics have the same influence on oxidative stress in cardiac surgery patients. Data are mean values ± SD. Pre-OP baseline, before induction of anaesthesia, intra-op immediately before termination of surgery, post-OP 24 h after surgery. (PDF 39 kb) [file 13054_2015_1082_MOESM1_ESM.pdf]
